# A Case Report of Duodenal Malignancy and Its Management With Pancreaticoduodenectomy

**DOI:** 10.7759/cureus.68915

**Published:** 2024-09-07

**Authors:** Vaishnavi D Rajurkar, Lokesh Singh Chauhan, Nisha D Barole, Shakti Sagar

**Affiliations:** 1 Pathology, Datta Meghe Institute of Higher Education and Research, Wardha, IND; 2 Pathology, Jawaharlal Nehru Medical College, Datta Meghe Institute of Higher Education and Research, Wardha, IND; 3 Clinical Research, Datta Meghe Institute of Higher Education and Research, Wardha, IND

**Keywords:** computed tomography, duodenum, malignancy, pancreaticoduodenectomy, whipple’s procedure

## Abstract

Gastrointestinal malignancies, most specifically duodenal malignancy, are uncommon in the population; however, they are tricky to manage because the lesions are diagnosed at a late stage and located in a complex area. This case report focuses on a patient who was diagnosed with a second (descending) part of the duodenum (D2) malignancy; the tumour was localised at the second part of the duodenum, and the management of this patient was through the Whipple procedure or pancreaticoduodenectomy. The patient complained of stinging abdomen pain.

Diagnostic examination comprised of CT and biopsy proved the presence of a malignant tumour originating from the D2 area. The tumour's characteristics and the patient's general health status are determined by using Whipple's procedure. Post-operative management entails the use of IV antibiotics, analgesics, multivitamins and nasojejunal (NJ) tube feeding. The case also expounds on the consequences of a multimodal treatment strategy, meticulous planning of the surgical procedure, and adequate post-operative management of D2 malignancies. These data may shed some light on the peculiarities of duodenal cancer management with the Whipple procedure; nonetheless, early diagnosis and proper management should remain the primary goals.

## Introduction

Cancers of the gastrointestinal tract (GIT), especially those arising from the duodenum, are technically demanding, given the region’s location and clinical characteristics [1]. Among these, the D2 malignancies, which are the tumours confined to the second part of the duodenum, are not frequent but are characterised by a high morbidity/mortality ratio due to a high potential for late diagnosis and hugely aggressive behaviour [2]. The duodenum is identified as the first segment of the small intestine. Slightly below the stomach, the duodenum, which is the second part of the small intestine, is approximately 25-30 cm long. The fact that the duodenum is a rather long and multiple ileal loops and its anatomical relationship with some critical structures, including the pancreas, bile duct, and mesenteric vessels, makes its diagnosis and surgery challenging [3]. Perhaps the most definitive therapy for duodenal malignancies, especially in the periampullary region, is the Whipple procedure, also termed pancreaticoduodenectomy; the operation was named after its discoverer Dr. Allen Oldfather Whipple in the 1930s [4]. It is worth pointing out that duodenal adenocarcinoma (DA) is much less frequent than other gastrointestinal (GI) cancers, yet it is the only primary duodenal malignancy. Another region of instruction that is important anatomically is the area of the second portion of the duodenum (D2). Cancer manifestations in the duodenum are usually symptomatic, and such symptoms are frequently vague and include abdominal pain, weight loss, and jaundice, thus resembling other gastrointestinal diseases [5].

Pancreaticoduodenectomy also termed as the Whipple surgery is the primary procedure to manage resectable duodenal cancers. This is a very delicate procedure that entails withdrawing the duodenum, part of the pancreas, the bile duct and sometimes part of the stomach then rearranging the bowels. Although this procedure may offer a chance of a cure, it becomes associated with important risks and should only be contemplated after careful selection of the right cases and in experienced hands [6]. Pancreaticoduodenectomy (PD), commonly referred to as the Whipple procedure to manage duodenal malignancy, is a multidisciplinary treatment plan that is preceded by a comprehensive work-up. This also entails the use of imaging scans such as computed tomography or magnetic resonance imaging to determine the size and location of the tumour as well as its proximity to other organs, as well as endoscopy for biopsy and additional tests. It requires coordination with surgeons and oncologists as well as radiologists to assess the probable necessity of neoadjuvant treatment for patients who have tumours that are potentially resectable [7].

Gastrointestinal malignancies account for 0.3%-1% of all cancers, with an incidence rate of less than 0.5 per 100,000 people [8]. Duodenum arises from the pyloric end of the stomach, is the most proximal section of the small intestine, and is the most commonly affected part [9]. Duodenal cancer, particularly of the adenocarcinoma type, is not a very common occurrence. As with several similar diseases, diagnosing this at the initial stages is impossible because the distinguishable signs are vague [10]. About the memo, it can be noted that the second part of the duodenum is the most common area for the occurrence of duodenal adenocarcinomas, which is approximately 0. The percentage of all malignant gastrointestinal tumours of this type accounts for only 3% [11]. In diagnosing and staging duodenal cancer, imaging studies are essential and include computed tomography (CT), magnetic resonance imaging (MRI), and endoscopic ultrasound (EUS). Histopathological confirmation requires a biopsy [12]. In these, the patient underwent comprehensive diagnostic evaluation, including imaging and biopsy, confirming a malignant tumour in the second portion of the duodenum.

## Case presentation

A 38-year-old female patient presented with a complaint of abdominal pain that was dull and aching. The pain was insidious in onset and gradually progressive, radiating to the back for the past two years. She did not experience nausea or vomiting, no history of fever or trauma, no bowel or bladder complaints, and no history of cough, cold or breathlessness during the onset of the disease. No history or significant family history of bowel cancer was reported. During a routine physical examination, she had better mental status and stable vital signs. Then, the patient was admitted. After admission, a contrast-enhanced CT (CECT) scan of the abdomen and pelvis was performed, which revealed a well-differentiated adenocarcinoma (WDACC) in the second and third portions of the duodenum (D2 and D3). It showed a well-defined adenocarcinoma intraduodenal mass measuring 4 x 3.4 cm involving the 2nd and 3rd part of the duodenum, compressing the ampulla and causing dilatation of the common bile duct (CBD). There was no evidence of metastatic disease. The liver showed a calcified hydatid cyst. The patient underwent eight cycles of chemotherapy before surgery. The patient underwent an eight-cycle chemotherapy regimen following a Whipple procedure for duodenal cancer, receiving Oxaliplatin 100 mg, Leucovorin 600 mg, and Florocid (5-FU) in doses of 600 mg and 3000 mg (Table 1).

**Table 1:** Chemotherapy chart

Cycle	Drugs	Dosage	Administration Route
1st	Oxaliplatin	100 mg	IV
	Leucovorin	600 mg	IV
2nd	Oxaliplatin	100 mg	IV
	DSY	500 ml	IV
3rd	Oxaliplatin	100 mg	IV
	Leucovorin	600 mg	IV
4th	Oxaliplatin	100 mg	IV
	Leucovorin	600 mg	IV
	Florocid	600 mg	IV
	Florocid	3000 mg	IV
5th	Oxaliplatin	100 mg	IV
	Leucovorin	600 mg	IV
	Florocid	600 mg	IV
	Florocid	3000 mg	IV
6th	Oxaliplatin	100 mg	IV
	Leucovorin	600 mg	IV
	Florocid	600 mg	IV
	Florocid	3000 mg	IV
7th	Oxaliplatin	100 mg	IV
	Leucovorin	600 mg	IV
	Florocid	600 mg	IV
	Florocid	3000 mg	IV
8th	Oxaliplatin	100 mg	IV
	Leucovorin	600 mg	IV
	Florocid	600 mg	IV
	Florocid	3000 mg	IV

After obtaining all essential fitness and preanesthetic check-ups, the patient underwent surgery under general anaesthesia with an epidural on 6 June 2024. The incision location was made in the bilateral sub-costal region. The surgery starts with resection of the gall bladder, duodenum, and proximal jejunum. The tumour was localized to the D2 region of the duodenum, with no evidence of disease spread to the liver, peritoneum or pelvis. There was no ascites. Hepatic flexure was brought down, and the duodenum was widely kocherized until the superior mesenteric vessels were exposed. The gastrocolic momentum was divided to enter the lesser sac, and respectability was assessed by tunnelling under the pancreatic neck and lifting it off the superior mesenteric vein. After ligating the right gastroepiploic and right gastric arteries, the stomach was divided. Cholecystectomy was then performed, and the portal structures, the common bile duct, portal vein and common hepatic artery were carefully identified, dissected and taped. The gastroduodenal artery was dissected, skeletonized, ligated, transfixed and divided. The common bile duct was later divided between stay sutures just above the insertion of the cystic duct. The jejunal loop, just beyond the duodenojejunal flexure, was mobilized in preparation for the resection of the specimen. The pancreatic neck was then divided with a sharp knife between stay sutures, ensuring perfect haemostasis of the pancreatic cut surface. To complete the resection, the uncinate process was dissected away from the right lateral border of the superior mesenteric vein.

**Figure 1:**
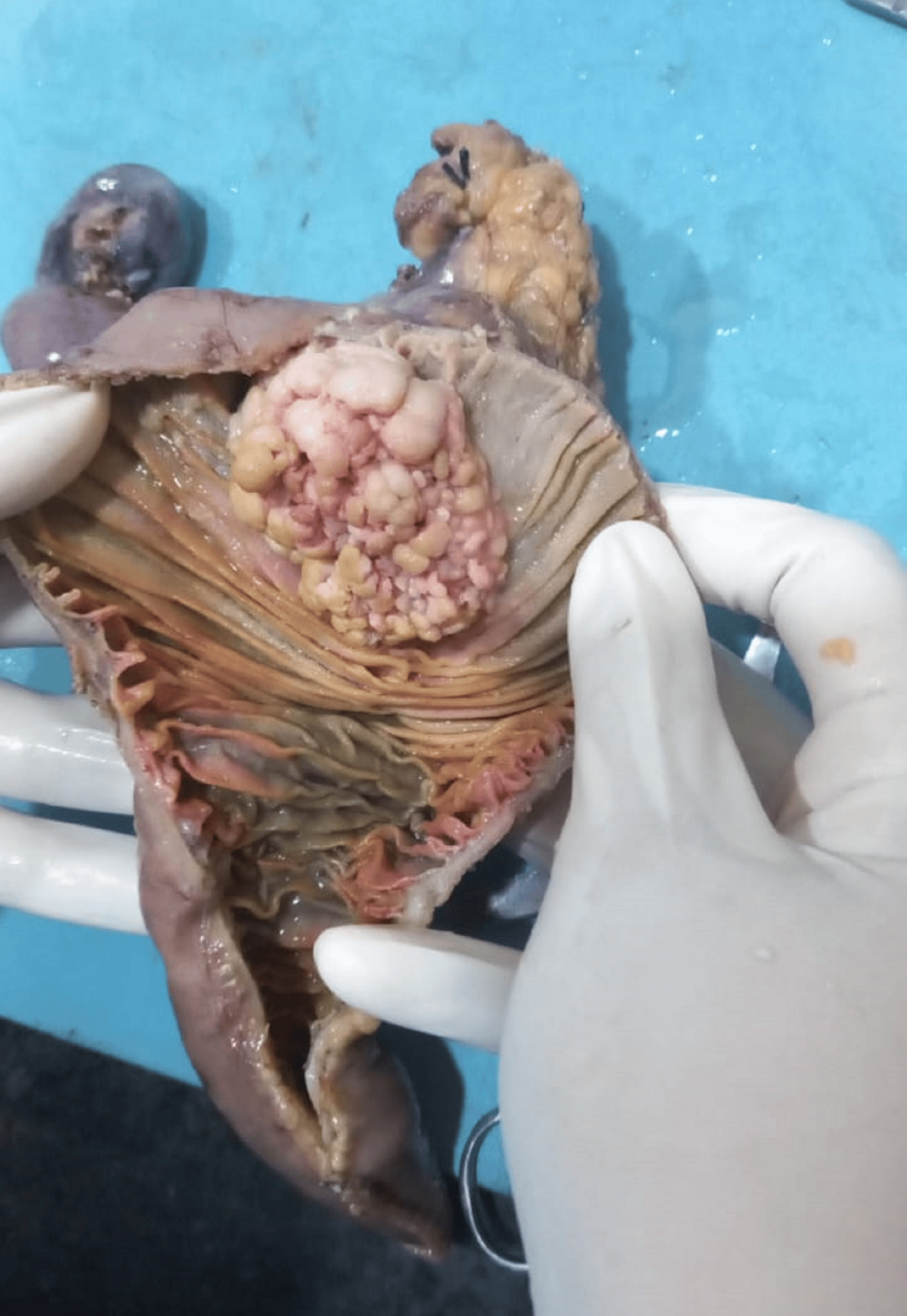
Cauliflower-like growth in the second part of the duodenum.

Whipple’s procedure, or pancreatojejunostomy, is a complex surgical operation used to treat cancers in the head of the pancreas and the duodenum, the first part of the small intestine. The procedure involves removing the tumour-containing portion of the duodenum, the head of the pancreas, part of the bile duct, the gallbladder, and sometimes part of the stomach. This extensive resection is necessary because of the close anatomical connections and potential cancer spread among these organs. During the surgery, a large abdominal incision is made, and nearby lymph nodes are also removed to check for cancer spread. Reconstruction involves connecting the remaining pancreas, bile duct, and stomach to the jejunum to maintain digestive function. Advantages of Whipple’s procedure include the potential cure for early-stage cancers, improved survival rates by removing the tumour and associated lymph nodes, symptom relief from issues like jaundice and pain, and comprehensive treatment that reduces the risk of local recurrence. During the pancreatojejunostomy procedure, the jejunum was brought up through a window in the mesocolon. The pancreatojejunostomy was performed using the dunking method. A choledochojejunostomy was executed with single-layer, interrupted 2-0 vicryl sutures. The gastrojejunostomy was completed in two layers with continuous 3-0 vicryl sutures. A nasojejunal (NJ) tube was inserted using a Freka tube. Haemostasis was confirmed, a drain was placed in the right upper quadrant, adjacent to the pancreatojejunostomy and choledochojejunostomy sites, and the incision was closed in layers. The entire specimen was sent for histopathological examination. After fixation, as shown in Figure 1, unifocal grey-white cauliflower-like growth in the 2nd and 3rd parts of the duodenum measured 4 x 3 cm.

**Figure 2:**
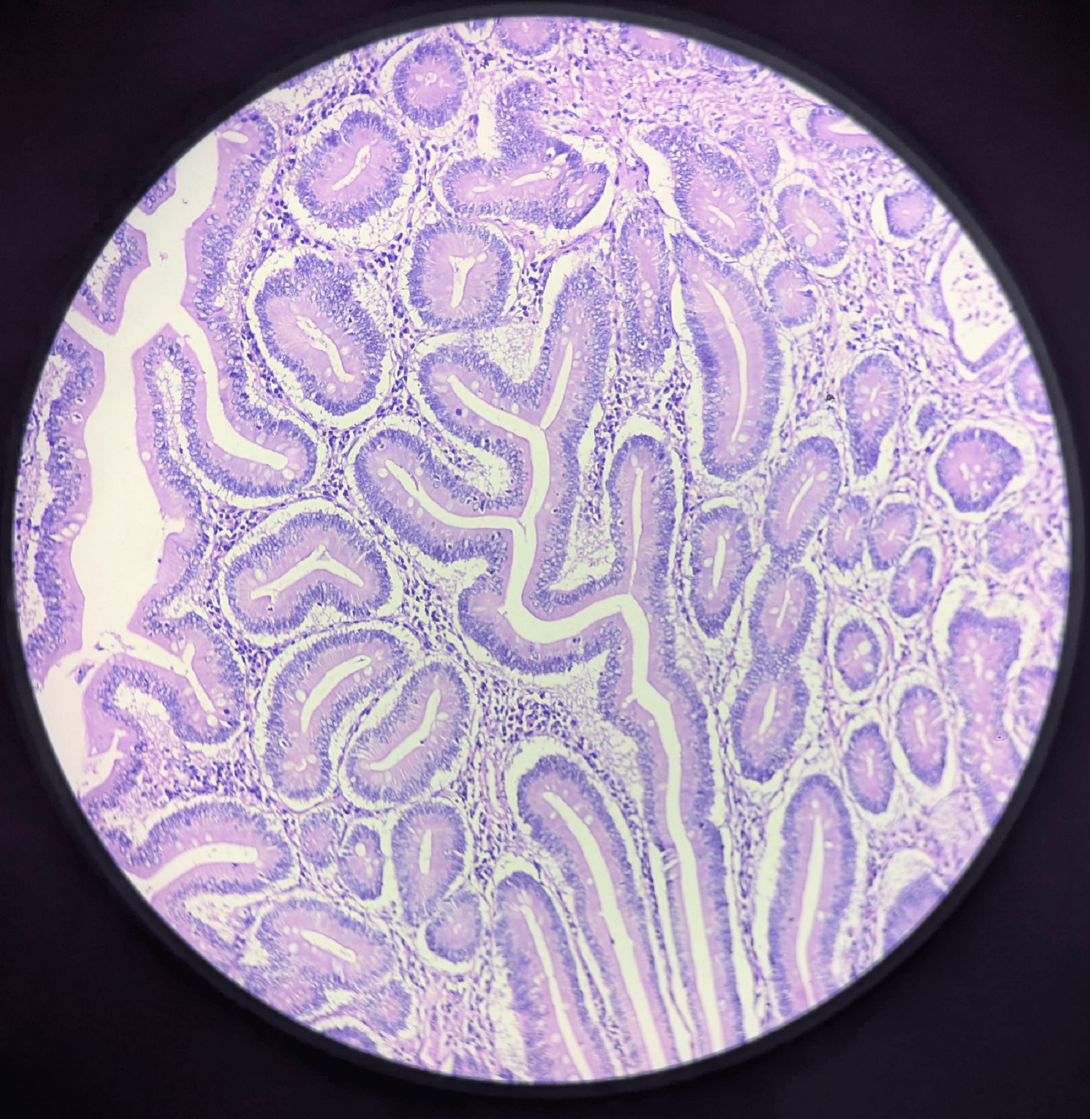
Well-differentiated adenocarcinoma (grade 1).

A well-differentiated adenocarcinoma (grade 1) extending up to muscular propria was seen on microscopy (Figure 2). Tumour necrosis was seen. There was no evidence of lymphovascular invasion or perineural invasion. All the specimen margins were negative for invasion. A total of 11 lymph nodes were identified from the various parts of the specimen and all were reported negative for metastasis. Based on these findings, this tumour was staged as yPT2N0Mx; American Joint Committee on Cancer Stage 1. Postoperatively, the patient was managed with IV antibiotics to prevent infections and IV analgesics for effective pain control, facilitating early mobilization. Nutritional support was provided through nasojejunal (NJ) tube feeding, with additional multivitamins to address any deficiencies. Fluid and electrolyte levels were closely monitored, and the care team remained vigilant for potential complications like pancreatic fistula and delayed gastric emptying. As the patient stabilized, a gradual transition to oral feeding was initiated to support recovery. The patient was discharged without any complications and is healthy.

## Discussion

The Whipple procedure, targeting cancers in the pancreatic head and duodenum, involves the removal of the pancreatic head, duodenum, gallbladder, bile duct, and sometimes part of the stomach, followed by reconstruction of the digestive tract through anastomoses like pancreatojejunostomy. This discussion emphasizes the role of PD in improving survival rates for patients with resectable tumours, acknowledging the technical challenges and risks involved. This broader perspective, addressing the reviewers' suggestions, ensures a more balanced and relevant analysis of the procedure [6]. The management of D2 malignancies through the Whipple procedure typifies the complexity and difficulty of managing cancers of the duodenum [2]. This case report highlights several important factors that are important to achieving successful outcomes in such complex cases. D2 malignancies are less common than oesophageal squamous cell carcinomas and adenocarcinomas, which can make diagnosis more difficult. The symptoms of duodenal cancers are normally vague and they can easily be mistaken for other gastrointestinal diseases thus causing a delay in diagnosis [13]. In this case, the patient had features such as abdominal pain, weight loss and jaundice which are generalized symptoms. This highlights the need for a high level of suspicion and the employment of advanced imaging modality and endoscopy in arriving at the correct diagnosis. Comprehensive diagnostic evaluation must be considered. Imaging modalities such as CT scans and MRI offer essential information on the tumour's size, location, and extension into adjacent structures. Endoscopic submucosal resection (ESU) and biopsy are applicable for histopathological diagnoses, providing absolute staging of the tumour and directing the evaluation of the treatment in all cases to guarantee an appropriate surgical intervention [14].

The Whipple procedure, per se, is a complex operation that demands high surgical skill and precision. The decision to proceed with this procedure is multi-parametric, considering the tumour's respectability, the status of the patient's physiology, and the likelihood of obtaining clear surgical margins. In the present case report, due to the complexity of the surgery, where several vital structures had to be resected and reconstructed, it was crucial to avoid intraoperative or postoperative problems such as delayed gastric emptying (DGE), pancreatic fistula, bleeding, intra-abdominal collections, and pulmonary issues [4]. Nevertheless, care after the surgery is also very important to the overall well-being of the patient. Delayed gastric emptying, pancreatic fistula, and infections for example are frequent and require careful monitoring and management. The surgery is extensive and affects the GIT, and therefore, nutritional supplementation is critical [15]. In this case, postoperative care was provided to the patient with attention given to complications that arose and strategies that could be used to foster quick recovery.

Consequently, the crucial point of a multidisciplinary approach in treating D2 malignancies should be underlined. Multidisciplinary teamwork with surgical, medical, radiology, gastro, and nursing specialities is beneficial regarding the patients’ conditions. Ideally, follow-up is required to check for throwbacks and complications that may arise in the long term [16]. Therefore, based on the findings of this case report, it may be concluded that D2 malignancies associated with the Whipple procedure are fraught with several difficulties. To enhance the prognosis, patients should be diagnosed as early as possible; surgery should be performed with exceptional regard to anatomical structures and postoperatively, patients should undergo thorough rehabilitation [17]. At the same time, it stresses the need to apply multisectoral cooperation precisely because of the challenges proposed, capitalised by D2 duodenal cancers. Neoadjuvant chemotherapy is increasingly used in managing pancreatic and duodenal cancers, especially in borderline resectable or locally advanced cases. Key benefits include downstaging tumours for easier resection, addressing micrometastatic disease early, and assessing tumour aggressiveness based on response. Common regimens include FOLFIRINOX or gemcitabine with nab-paclitaxel, tailored to the patient's condition. This approach can simplify surgery and reduce complications by minimizing vascular involvement and tumour size [18].

## Conclusions

The histopathology report diagnosed the patient with a well-differentiated adenocarcinoma, with no evidence of metastatic lymph nodes, and the tumour was staged as yPT2N0Mx. The patient subsequently underwent a Whipple procedure. The case underscores the complexities of managing D2 duodenal malignancies and highlights the importance of early diagnosis, precise surgical intervention, and comprehensive postoperative care. The successful Whipple procedure in this patient demonstrates the necessity of advanced imaging, meticulous surgical technique, and diligent postoperative management.
